# Chemoprevention of Colon Cancer by DFMO, Sulindac, and NO-Sulindac Administered Individually or in Combinations in F344 Rats

**DOI:** 10.3390/cancers15154001

**Published:** 2023-08-07

**Authors:** Venkateshwar Madka, Jagan M. R. Patlolla, Karthikkumar Venkatachalam, Yuting Zhang, Gopal Pathuri, Nicole Stratton, Stanley Lightfoot, Naveena B. Janakiram, Altaf Mohammed, Chinthalapally V. Rao

**Affiliations:** 1 Center for Cancer Prevention and Drug Development, Department of Internal Medicine, Hem-Onc Section, Stephenson Cancer Center, University of Oklahoma Health Sciences Center, Oklahoma City, OK 73104, USA; vmadka@ouhsc.edu (V.M.);; 2 Translational Research Program, Division of Cancer Treatment and Diagnosis, National Cancer Institute, Bethesda, MD 20892, USA; 3 Chemopreventive Agent Development Research Group, Division of Cancer Prevention, National Cancer Institute, Rockville, MD 20850, USA; 4 VA Medical Center, Oklahoma City, OK 73104, USA

**Keywords:** colorectal cancer, chemoprevention, NSAIDs, polyamines, nitric oxide

## Abstract

**Simple Summary:**

Colon cancer is a major health problem, and its occurrence is rising particularly among young adults. Preventing this cancer is urgently needed. Earlier studies in animal models as well as on human have shown promising preventive effect of agents like Sulindac and DFMO. Unfortunately, the long-term use of these agents at high doses is associated with some side effects, thus alternative strategies are being explored to employ these drugs for preventing CRC. In the present study, we used approaches such as (i) Combination of low dose of the two agents, (ii) testing the efficacy of sulindac derivatives. For this colon cancer was chemically induced in rats, later the test agents, Sulindac, NO-Sulindac and DFMO were administered to the rats, either individually or in combination. At the end of the study, we observed that these treatment regimens inhibited the tumor progression to advanced stage with no evidence of toxicity. Thus our study demonstrate that low dose combination of these agents may be a promising approach to use them for colon cancer prevention without causing any toxicity.

**Abstract:**

Non-steroidal anti-inflammatory drugs (NSAIDs) are promising colorectal cancer (CRC) chemopreventive drugs; however, to overcome NSAIDs’ associated side effects, there is a need to develop safer and efficacious approaches. The present study was designed to evaluate (i) the efficacy of nitric-oxide releasing (NO)-Sulindac as compared to Sulindac; (ii) whether NO-Sulindac is superior to Sulindac in enhancing low-dose difluoromethylornithine (DFMO)-induced chemopreventive efficacy, and (iii) assessing the key biomarkers associated with colon tumor inhibition by these combinations. In F344 rats, colonic tumors were induced by azoxymethane (AOM). At the adenoma stage (13 weeks post AOM), groups of rats were fed the experimental diets containing 0 ppm, 500 ppm DFMO, 150 ppm Sulindac, and 200 ppm NO-Sulindac, individually or in combinations, for 36 weeks. Colon tumors were evaluated histopathologically and assayed for expression levels of proliferative, apoptotic, and inflammatory markers. Results suggest that (except for NO-Sulindac alone), DFMO, Sulindac individually, and DFMO combined with Sulindac or NO-Sulindac significantly suppressed AOM-induced adenocarcinoma incidence and multiplicities. DFMO and Sulindac suppressed adenocarcinoma multiplicity by 63% (*p* < 0.0001) and 51% (*p* < 0.0011), respectively, whereas NO-Sulindac had a modest effect (22.8%, *p* = 0.09). Combinations of DFMO plus Sulindac or NO-Sulindac suppressed adenocarcinoma incidence (60%, *p* < 0.0001; 50% *p* < 0.0004), and multiplicity (81%, *p* < 0.0001; 62%, *p* < 0.0001). Rats that were fed the combination of DFMO plus Sulindac showed significant inhibition of tumor cell proliferation and induction of apoptosis. In addition, enhancement of p21, Bax, and caspases; downregulation of Ki-67, VEGF, and β-catenin; and modulation of iNOS, COX-2, and ODC activities in colonic tumors were observed. These observations show that a lower-dose of DFMO and Sulindac significantly enhanced CRC chemopreventive efficacy when compared to NO-Sulindac alone, and the combination of DFMO and NO-Sulindac was modestly efficacious as compared to DFMO alone.

## 1. Introduction

Colorectal cancer (CRC) is one of the leading causes of cancer-related deaths in the US and worldwide [1]. Over the past few decades, non-steroidal anti-inflammatory drugs (NSAIDs) have been widely studied for cancer chemopreventive properties, including CRC. Sulindac is one of the most widely studied NSAIDs in human clinical trials of high-risk CRC patients [2]. Preclinical and clinical studies have demonstrated uniformly chemopreventive properties of Sulindac in CRC [3,4,5]. Sulindac usage is beneficial from the efficacy standpoint, but has been shown to carry significant gastrointestinal toxicities in long term use [6,7]. Thus, a different mode of the action exerted by a low-dose combination of agents represents a practical approach for improving the chemopreventive efficacy and eliminating unwanted toxicities. In order to overcome the limitations of gastrointestinal (GI) toxicities, over the past decade, two major approaches were followed: (i) use of a low-dose combinational approach, where a low-dose sulindac combined with difluoromethylornithine (DFMO) has shown dramatic colon tumor inhibitory effects; and (ii) developing novel derivatives of Sulindac by chemically modifying the parent molecule to overcome GI toxicities and vascular bleeding.

**Figure 1:**
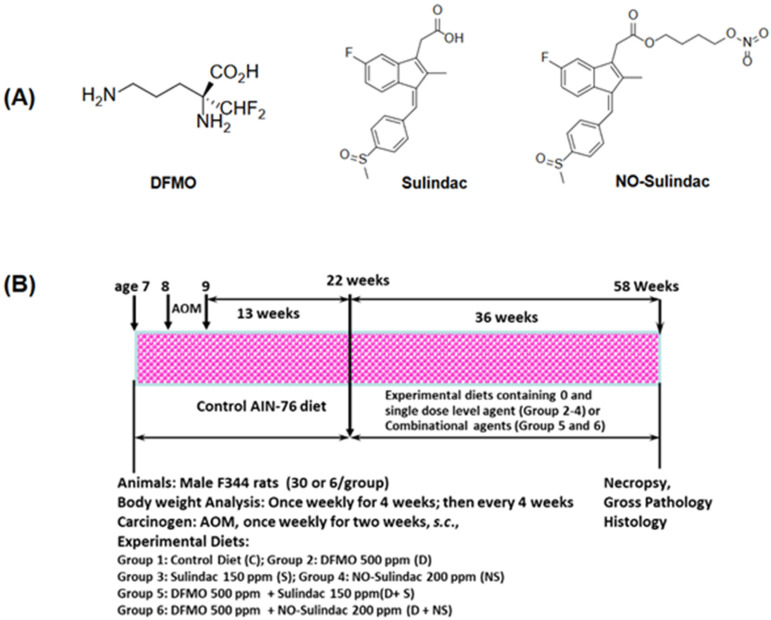
Experimental design and structure of agents tested. (**A**) Chemical structures of chemopreventive agents tested—DFMO, Sulindac, and NO-Sulindac. (**B**) Experimental protocol for the evaluation of chemopreventive efficacy of DFMO, Sulindac and NO-Sulindac in a rat colon cancer model. Colon tumor efficacy assay: Groups of rats (36 rats/group; 30 AOM + 6 Vehicle) were fed the control diet (AIN 76A) at age 7 weeks; rats were given 15 mg azoxymethane/Kg body weight once weekly for 2 weeks. Thirteen weeks after second AOM injection (adenoma stage), rats were fed the control diet and experimental diets containing 500 ppm DFMO, 150 ppm Sulindac, 200 ppm NO-Sulindac, and combinations of 500 ppm DFMO plus 150 ppm Sulindac or 500 ppm DFMO plus 200 ppm NO-Sulindac for 36 weeks to assess colorectal adenocarcinomas (detailed information has been given in Section 2).

Various derivatives of nitric-oxide-donating Sulindac (NO-Sulindac) and phospho-Sulindac (P-Sulindac) have been designed and developed to overcome GI toxicities. NO-Sulindac, like other NO–donating NSAIDs (NO-NSAID), consists of a conventional NSAID which bears a covalently attached moiety that ultimately releases NO (Figure 1A). The rationale for their development was that the NO that they release would compensate for the inhibition of prostaglandin synthesis by its NSAID moiety; the mechanism of their apparent gastro-protection seems more complex. Similarly, the mechanism of their enhanced potency in vitro has not been fully explored in the in vivo models. So far, published data suggest that NO-releasing NSAIDs carry the promise of higher potency and greater safety compared with their conventional counterparts [8]. In vitro data have shown that NO-NSAIDs have been invariably more potent than their parent compounds, although their enhanced potency ranged between 10- to >1000-fold in in vitro studies [9]. Animal studies have shown that NSAIDs, including NO-NSAIDs, suppress the formation of AOM-induced colonic aberrant crypt foci and intestinal polyposis in *Min* mice [10,11,12]. Importantly, NO-aspirin and NO-Indomethacin suppressed AOM-induced colon adenocarcinomas in rats by 60–80% without any observable toxicity [13]. Although in vitro studies support the NO-Sulindac superiority in cancer cell lines compared to Sulindac, its relative potential in well-established animal models, such as carcinogen azoxymethane (AOM) injected Fisher 344 rats, which develop CRC recapitulating important features of human CRC, was not studied [14]. The AOM CRC model system has been highly informative in assessing potential chemopreventive agents and their further development in human clinical trials.

The two most commonly mutated genes in human CRC are *APC* and *K-RAS* [15,16], and the downstream target for these genes has been shown to independently activate polyamine synthesis [17]. In polyamine synthesis, ornithine decarboxylase (ODC) enzyme activity is very high in colonic tumors as compared to normal-appearing adjacent colonic mucosa [18,19]. With increased emphasis on the use of a combination chemoprevention approach [20], research using (DFMO), which is a specific ODC inhibitor, plus Sulindac, which stimulates polyamine acetylation and acts as an anti-inflammatory agent, was evaluated in Apc^Min/+^ mouse models [21,22], as well as in clinical trials of sporadic colorectal adenomas [23].

Previous studies from our laboratory used 2000 to 4000 ppm of DFMO (~2000 mg and ~4000 mg/day HED) and ~160 to 320 ppm of Sulindac in the diet for our colon cancer studies [3,24]. In the present study, we de-escalated the doses to 500 ppm DFMO (500 mg/day HED) and 150 ppm (150 mg/day HED) of Sulindac in the diet. This study also assesses the chemopreventive efficacy of NO-Sulindac, which promises to be devoid of the unwanted side effects of parent molecule Sulindac, but has the same anti-inflammatory properties with additional tumor inhibitory effects as NO. In addition, we evaluated the combination of NO-Sulindac and DFMO to know if it possesses greater efficacy when compared to the combination of DFMO and Sulindac. In addition to their chemoprevention efficacy, we assessed important aspects of the mechanism of action of these compounds in combinations and their effect on molecular targets related to colon carcinogenesis, including inflammation, proliferation, and apoptosis.

## 2. Materials and Methods

Animals, Diets, and Chemopreventive Agents. All animal experiments were carried out in accordance with NIH guidelines and University of Oklahoma Health Sciences Center Institutional Animal Care and Use Committee (IACUC) approved protocol. Male F344 rats were obtained at six weeks of age from Envigo Laboratories. Ingredients for the semi-purified diets were irradiated and purchased commercially (Bio-serv, Flemington, NJ, USA). Diets were modified based on the American Institute of Nutrition (AIN-76A diet) reformulation. The diet included 20% casein, 52% corn starch, 13% dextrose, 5% corn oil, 5% alphacel, 3.5% AIN mineral mix, 0.3% *D*,*L*-methionine, and 0.2 choline bitartrate [25]. DFMO, Sulindac, and NO-Sulindac (Figure 1A) were provided by the National Cancer Institute chemopreventive drug repository. Each drug was premixed with a small quantity of casein, then blended into a bulk diet using a Hobart Mixer. Both control and experimental diets were prepared weekly and stored in a cold room. The agent(s) content in the experimental diets was determined periodically in multiple samples taken from the top, middle, and bottom portions of individual diet preparations in order to verify uniform distribution. AOM (CAS:25843-45-2) was purchased from MRI Global (Kansas City, MO, USA).

Dose Selection. The experimental protocol is summarized in Figure 1B. Based on earlier studies, dose levels were de-escalated, and single low-dose levels of 500 ppm (mg/kg diet) DFMO, 150 ppm Sulindac, 200 ppm NO-Sulindac, and low-dose combinations of 500 ppm DFMO plus 150 ppm Sulindac and 500 ppm DFMO plus 200 ppm NO-Sulindac were selected, considering these doses are safe for long-term administration [3,13].

CRC Bioassay. A total of 216 male F344 rats, received at weaning, were acclimatized for 7 days and had unrestricted access to a modified AIN-76A control diet. Following acclimatization, all rats were randomly distributed by weight into various groups (36 rats/group; Figure 1B). They were housed in ventilated cages with filter tops (3 rats/cage) under controlled conditions with a 12 h light and dark cycle at 50% relative humidity and 21 °C. At 8 weeks of age, animals intended (30 AOM-treated) for carcinogen treatment received 2 weekly s.c. injections of AOM (15 mg/kg body weight). In addition, groups (6 rats/group) given control and experimental diets were given an equal volume of normal saline instead of AOM. After the second injection of AOM or normal saline, rats were placed on control diet until 22 weeks of age; then, experimental diets containing DFMO, Sulindac, NO-Sulindac, and their combinations were fed to respective groups until termination. Body weights were recorded every 2 weeks until the 16th week, and then every 4 weeks until termination of the experiment at 50 weeks after the last AOM treatment. At the termination, all organs, including the intestine, were examined grossly under the dissection microscope. Colon tumors with a diameter ≥0.5 cm were cut into halves; one half was quickly frozen in liquid nitrogen and stored at −80 °C until analyzed for ODC activity, and for the expression levels of proliferative, apoptotic, and inflammatory markers. The remaining portions of tissues were fixed in 10% neutral buffered formalin and processed by standard methods for histopathological evaluation [14,26].

Serum Analysis. Whole blood collected at termination was allowed to coagulate and was processed to prepare serum by centrifugation. Frozen serum samples were allowed to thaw and used to determine the liver and kidney toxicity parameters. Aspartate transaminase (AST), alanine aminotransferase (ALT), creatinine (CREA), and blood urea nitrogen (BUN) were evaluated using an IDEXX Catalyst instrument following manufacturer’s instructions.

Immunohistochemistry (IHC). Effects of DFMO, Sulindac, and NO-Sulindac administered individually or in combination on tumor cells were evaluated using IHC analysis of formalin-fixed paraffin embedded tissue sections (FFPE). Biomarkers evaluated were Ki67, β-catenin, VEGF, and Cyclin D1 using standard IHC procedures. Briefly, paraffin-embedded colonic tissues from different treatment groups were cut into 5 µm thick sections and mounted on microscopic slides. After deparaffinization, sections were blocked for endogenous peroxidase quenching activity and incubated with a 1% blocking solution. Biotinylated primary antibody was applied at appropriate dilution (1:100–1:300) overnight at 4 °C. The next day, slides were washed in PBS and incubated with HRP-tagged secondary antibody for 2 h, then rinsed with PBS. After rinsing, the slides were incubated with the chromogen 3,3′-diaminobenzidine (DAB) for 3 min, then rinsed and counterstained with hematoxylin. Cells with a brown staining were considered positive. Digital images of multiple fields were analyzed using ImageJ IHC profiler (IHC Profiler: an open-source plugin for the quantitative evaluation and automated scoring of immunohistochemistry image) to quantify and compare the marker expressions between treated and untreated rat tumors, following methods published earlier [25,27].

Effect of Chemopreventive Agents on Colonic Tumor ODC, iNOS, and COX-2 Enzyme Activities and Polyamines. Measurement of ODC, iNOS, and COX-enzymes activities were carried out by previously published procedures using [^14^C]-ornithine, *L-*[^3^H]-arginine, and ^14^C-arachidonic acid as substrates [13]. *Polyamine analysis:* Each colonic mucosal and tumor sample was suspended in ice-cold PBS and homogenized on ice using a Polytron Homogenizer (Brinkman Instruments, Westbury, NY, USA). Protein-free supernatant (500 µL) was mixed with 350 µL saturated sodium carbonate and 400 µL of 37 mmol/L dansyl chloride, then the mixture was incubated at 60 °C for 1 h. Dansylated polyamines were extracted in toluene, dried, then redissolved in 100 µL of acetonitrile, and finally quantified by reverse-phase HPLC method. Briefly, a chromatography procedure using a Shimadzu C_18_, 33 × 4.6 mm i.d. cartridge column with 10 mmol/L heptane sulfonate buffer, pH 3.4, in acetonitrile gradient at a flow rate of 1.5 mL/min were applied, and dansylated polyamines were detected using fluorescence detection set for excitation at 330 nm and emission at 470 nm in Shimazu fluorescence detector 510.

Western Blot. Polyclonal antibodies for p21, Bax, NRF2, and Caspases 6 and 8 were procured from Abcam (Waltham, MA, USA) or Cell Signal Technology (CST) (Danvers, MA, USA). Tumors harvested from different groups were lysed in ice-cold RIPA buffer (Thermo Scientific, Waltham, MA, USA), and Halt™ Protease and Phosphatase Inhibitor Cocktail (Thermo Scientific, USA) and the protein content was determined by using the Bio-Rad Protein Assay reagent (Hercules, CA, USA). Separation of proteins (50 μg) was resolved on an SDS-PAGE and transferred onto PVDF membranes. Membranes were blocked with a solution containing 10 mmol/L Tris-HCl (pH 7.5), 150 mmol/L NaCl, 0.01% (*v*/*v*) Tween 20, and 5% BSA, and incubated overnight with either anti-p21 (ab109199; 1:1000), anti-Bax (CST #2772; 1:1000), anti-NRF2 (CST #12721; 1:1000), anti-Caspase6 (ab185645; 1:1000), anti-Caspase8 (ab25901; 1:1000), or anti-β-actin (1:1000). After washing the blots in TBST, the blots were incubated with horseradish peroxidase-conjugated secondary antibody corresponding to primary antibody followed by washing with TBST. Washed blots were incubated with Super Signal West Pico Chemiluminescence Substrate (Pierce, Rockford, IL, USA) and images were captured using a Gbox instrument (Syngene, Bangalore, India) [25]. The PVDF membranes were stripped with Restore™ PLUS Western Blot Stripping Buffer (Thermo Fisher #46430) and reprobed with additional antibodies following the blocking, then the images were captured as aforementioned.

Statistical Analysis. All results are expressed as Mean ± SE. Differences in body weights among groups were analyzed by ANOVA. Tumor incidences (percentage of rats with tumors) among different groups were compared by the Fisher’s Exact test. Tumor multiplicity (number of tumors per rat), organ weights, serum parameters, and protein expression and activities were analyzed by unpaired *t*-test with Welch’s correction. Differences were considered statistically significant at *p* < 0.05.

## 3. Results

**Figure 2:**
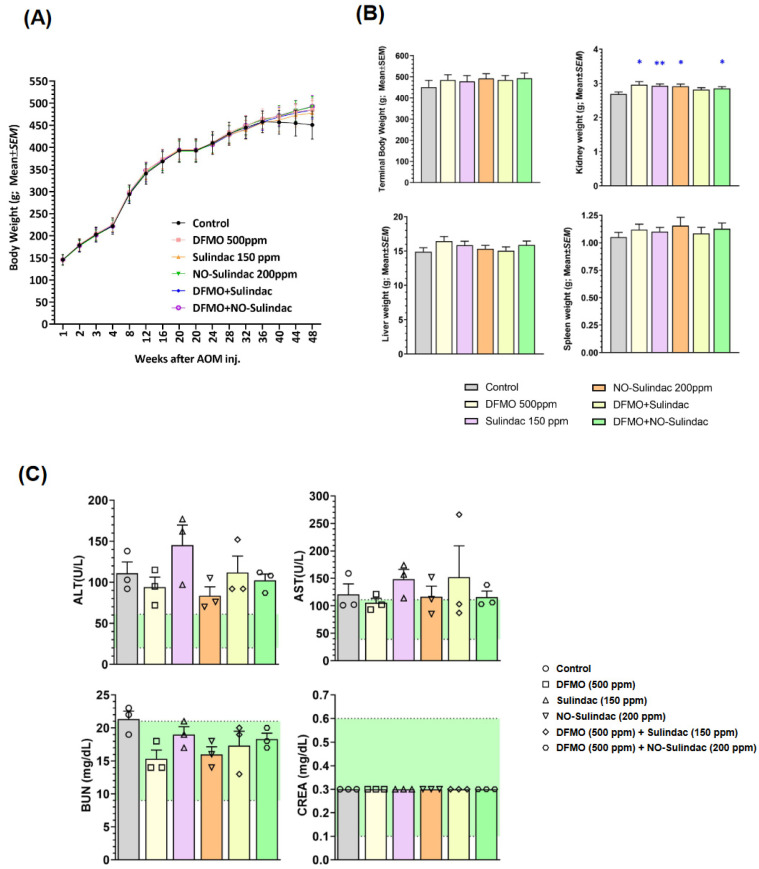
Toxicity evaluation of chronic chemopreventive agent treatment in rats. (**A**) Weekly body weight gain on the rats treated with various chemopreventive agents individually and in combination. (**B**) Comparison of the body weights and organ weights (Mean ± SEM) at termination. (**C**) Serum level of biomarkers of liver function (AST, ALT) and kidney function (BUN, CREA), *n = 3*. Normal range for each parameter as provided by IDEXX instrument is denoted by green shaded region. Significance analyzed using Student’s *t*-test. * = *p* < 0.05; ** = *p* < 0.01.

General Observations. DFMO, Sulindac, and NO-Sulindac administered alone or in combination to male F344 rats, for the experimental period, showed no significant effect on their body weights. During 36 weeks of administration of the above compounds in the diet, there were no apparent adverse effects on the rats. The body weights of all rats that were fed diets of different groups were comparable with those of the corresponding control groups throughout the study (Figure 2A). Chronic administration of DFMO, Sulindac, and NO-Sulindac, individually or together, produced no outward signs of toxicity (Figure 2B,C). Chronic administration of NO-Sulindac did not produce any gastrointestinal erosions or other signs of toxicity, nor any gross changes indicative of toxicity in several organs that were examined. However, two rats in the AIN-76A control diet and DFMO group, and one rat from both Sulindac and NO-Sulindac, developed ear duct tumors. These moribund rats were euthanized 4 weeks before the scheduled termination. Apart from that, AOM-alone-treated rats showed a decrement in their kidney weight, which might be the tumor burden caused by AOM.

**Figure 3:**
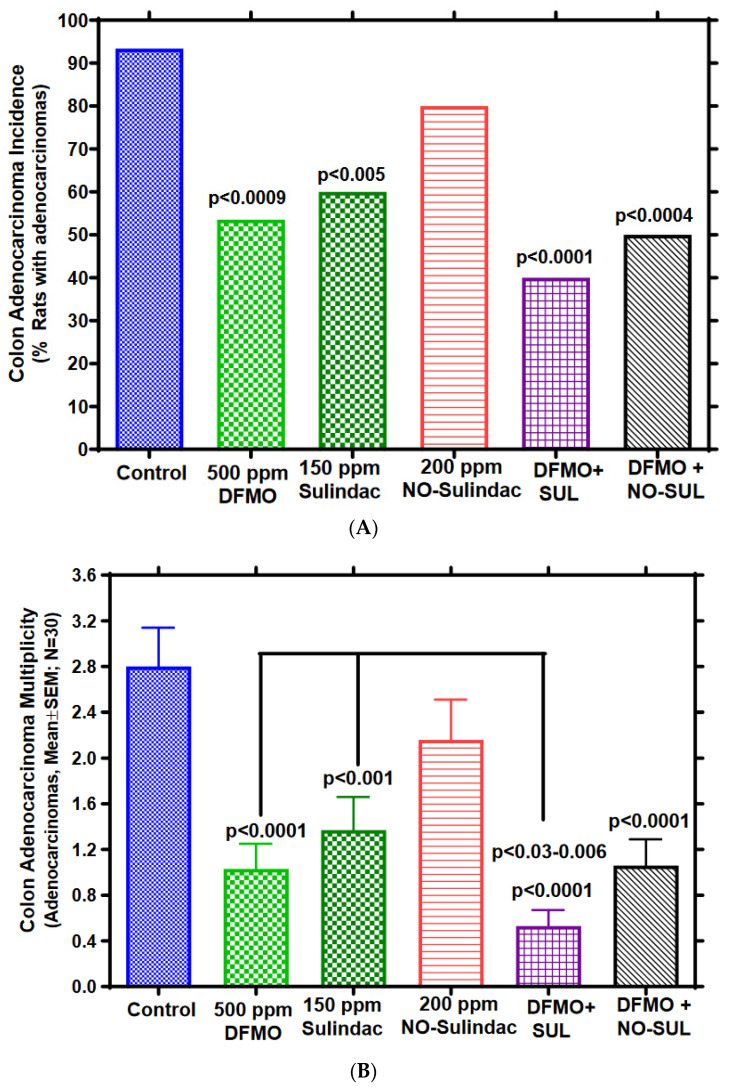
Efficacy of chemopreventive agents against AOM-induced CRC in F344 rat model. (**A**) Effect of DFMO, Sulindac, or NO-Sulindac alone and/or in combination on AOM-induced colon adenocarcinoma incidence (percentage of rats with colon adenocarcinomas) in rats. Statistical significance between control and treatment groups for carcinoma incidence was analyzed by Fisher’s Exact test. (**B**) Effect of DFMO, Sulindac, or NO-Sulindac alone or in combination on AOM–induced colon adenocarcinoma multiplicity (mean adenocarcinomas/colon) in rats. Number of colon adenocarcinomas (mean ± SEM) in rats, administered control and experimental diets containing 500 ppm DFMO, 150 ppm Sulindac, 200 ppm NO-Sulindac individually or in combinations at adenoma stage intervention (23 weeks of age). Adenocarcinoma multiplicity significance between control and treatment groups were analyzed by *t*-test with Welch’s correction, *n* = 30.

The combination of DFMO and Sulindac significantly inhibited the incidence and multiplicity of colon adenocarcinomas. Colonic tumors were classified by histopathology as adenomas (benign tumors with dysplastic glandular epithelium) or adenocarcinomas (malignant tumors with well or moderately differentiated gland formation) (3, 14). Most of the colonic tumors in this experiment were adenocarcinomas (~80%), predominately tubular adenocarcinomas, and fewer tumors were signet-ring mucinous adenocarcinomas. The majority of the colonic tumors (~80%) originated in the distal region (Figure S1). As shown in Figure 3A,B and Table 1, AOM–treated rats fed control diet had 93.3% adenocarcinoma incidence (percentage of rats with colon adenocarcinomas) and multiplicity (number of adenocarcinomas/colon) of 2.8 ± 0.34 (mean ± SEM). On the other hand, administering DFMO at 500 ppm to AOM-treated rats inhibited colon adenocarcinoma incidence by 42.8% (*p* < 0.001) and multiplicity by 63% (*p* < 0.0001); Sulindac at 150 ppm inhibited colon adenocarcinoma incidence by 35.7% (*p* < 0.005) and multiplicity by 51% (*p* < 0.0011), whereas NO-Sulindac at 200 ppm inhibited colon adenocarcinoma incidence by 14.25% and multiplicity by 22.8% (not significant; NS). In the combination groups, the adenocarcinoma incidence inhibition in DFMO plus Sulindac was 57.1% (*p* < 0.0001) and multiplicity inhibition was 81% (*p* < 0.0001), whereas in the DFMO plus NO-Sulindac group, the total adenocarcinoma incidence inhibition was 46.4% (*p* < 0.0004) and multiplicity inhibition was 62.1% (*p* < 0.0001). Importantly, as compared to DFMO or Sulindac alone, the combination of DFMO + Sulindac had a significant synergistic inhibitory effect (48.5% to 58.5%, *p* < 0.03–0.006) on colon adenocarcinoma multiplicity. However, the colon adenoma incidence and multiplicities were not significantly impacted by various treatments (Table 1).

Effect of Chemopreventive Agents Alone or in Combination on Colon Adenocarcinoma iNOS, COX-2, ODC Activities, and Polyamine levels. Table 2 summarizes the effect of DFMO, Sulindac, NO-Sulindac, or their combinations on the activities of iNOS, COX-2, and ODC in colon adenocarcinomas. DFMO and Sulindac significantly suppressed rat colon tumor iNOS (*p* < 0.001–0.005), and their combination showed greater inhibitory effect (*p* < 0.0001) as compared to individual administration alone, whereas NO-Sulindac failed to show any significant effect (*p* = 0.2) on iNOS activity in colonic tumors, nor provided additional inhibitory effect with DFMO combination. DFMO, Sulindac, NO-Sulindac, DFMO + Sulindac, and DFMO + NO-Sulindac inhibited colon adenocarcinoma COX-2 activities by 27.6% (*p* = 0.051), 45.4% (*p* < 0.002), 24.3% (*p* = 0.07), 55% (*p* < 0.0001), and 30.2% (*p* < 0.05), respectively. Sulindac alone and its combination with DFMO have a strong inhibitory effect on the PGE_2_ formation. With regard to colonic tumor ODC activities, as anticipated, only DFMO or its combinations showed a significant inhibitory effect. However, Sulindac and NO-Sulindac had modest but not significant inhibitory effects. Polyamines analyzed for putrescine, spermidine, and spermine (Table 2) clearly suggest DFMO alone had significant inhibitory effects, whereas Sulindac had modest effects. Overall, polyamine data clearly correlate with ODC activity and tumor inhibition induced by DFMO alone or in combination with Sulindac.

**Figure 4:**
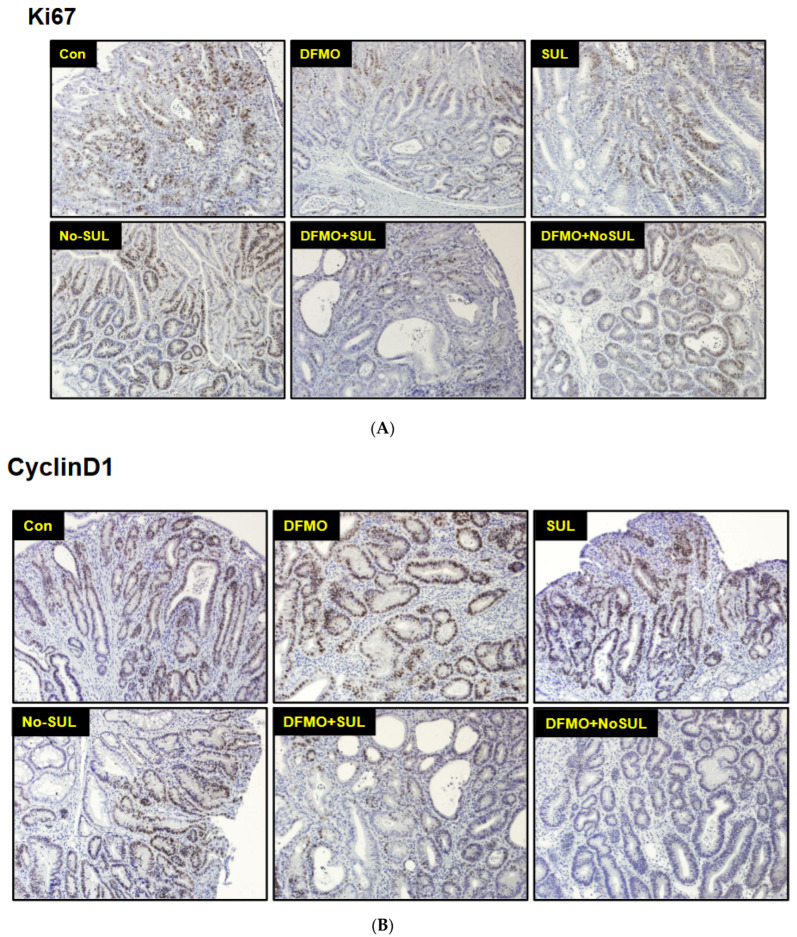

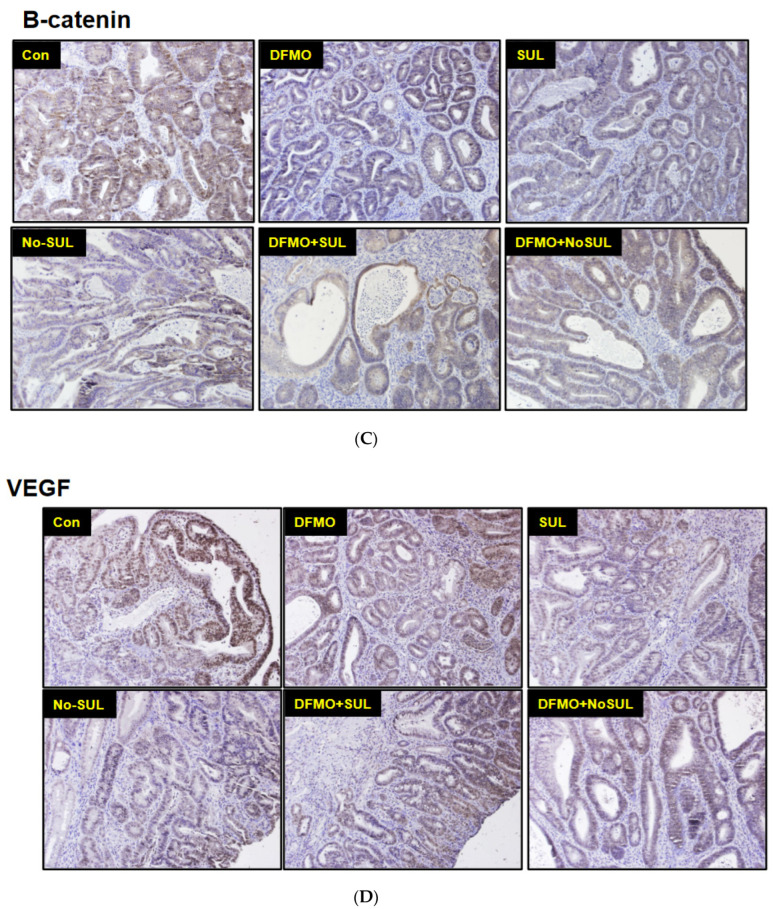

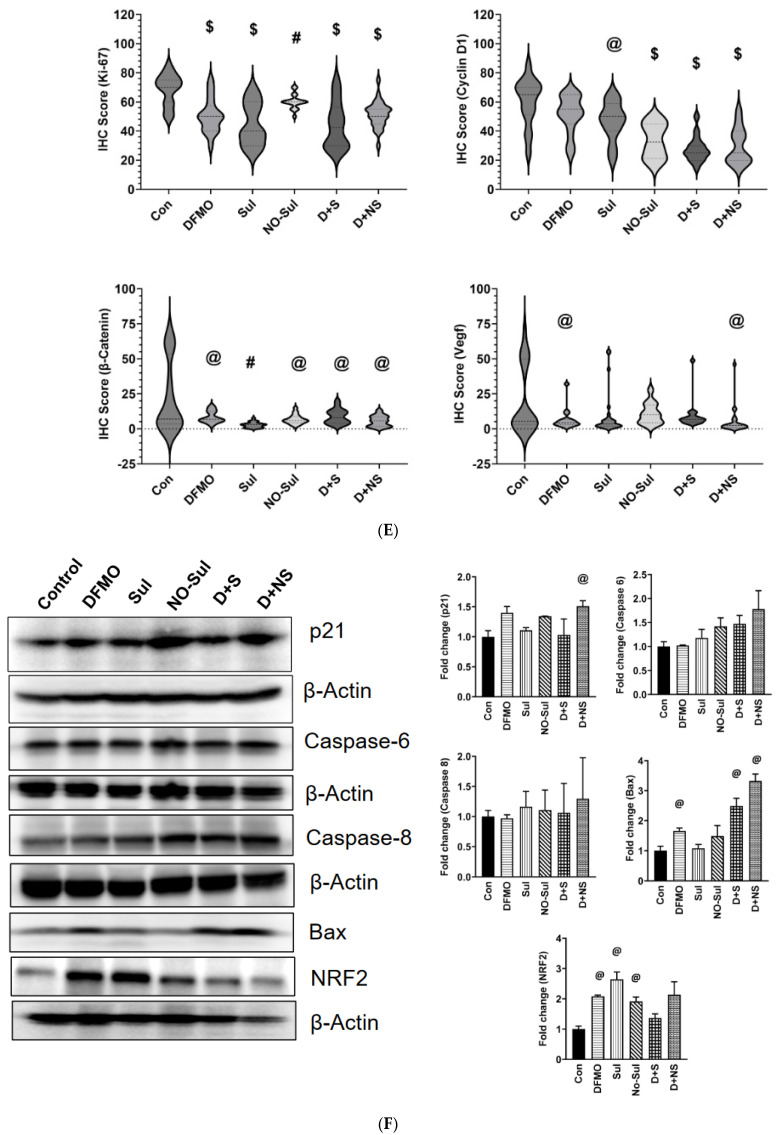
Analysis of the biomarkers of cell proliferation and apoptosis using Immunohistochemical staining and Western blotting. Formalin-fixed paraffin-embedded tissue sections were processed and expression of Ki-67, cyclin D1, β-Catenin, and VEGF were evaluated using IHC (**A**–**D**), *n* = 3. Digital images were analyzed using ImageJ IHC profiler to derive IHC score for comparison (**E**), *n* = 3. Frozen tumor samples were used for protein expression analysis using Western blot analysis (**F**), *n* = 2. Colonic tumors of rats administered 0 ppm, 500 ppm DFMO, 150 ppm Sulindac (Sul), 200 ppm NO-Sulindac (NO-Sul) alone and/or combination of 500 ppm DFMO plus 150 ppm Sulindac (D + S), or 500 ppm DFMO plus 200 ppm NO-Sulindac (D + NS) were collected at termination. Colonic tumors were either fixed in formalin or snap-frozen and stored at −80 °C for biomarker analysis. Control and drug-treated rat colon tumor tissues were homogenized in protein lysis buffer and were subjected to SDS-PAGE. Membranes were probed with specific primary antibodies and then peroxidase-conjugated appropriate secondary antibodies. Proteins were visualized with enhanced chemiluminescence detection system. Densitometric analysis of protein bands was done using ImageJ and normalized to loading control. Student’s *t*-test with Welch correction was used to identify statistically significant differences. @ = *p* < 0.05; # = *p* < 0.01; $ = *p* < 0.0001. See Figure S2 for original Western Blots.

Effect of Chemopreventive Agents’ Treatment on Tumor Cell Proliferation and Apoptosis. IHC analysis of the tumor sections indicated significant reduction in Ki67 and cyclin D1 positive tumor cells in the chemopreventive-agent-treated groups when compared to control group tumors (Figure 4), indicating tumor efficacy is partly driven by suppression of tumor cell proliferation. Expression level of p21^waf1/cip1^ is an important indicator of colonic tumor growth arrest and apoptosis. There was limited expression of p21^waf1/cip1^ in tumors from rats fed control diet. Induction of p21^waf1/cip1^ expression was observed in all groups fed with diet containing chemopreventive agents, but it was induced more in colonic tumors from rats fed DFMO at 500 ppm alone or in combination of DFMO at 500 ppm plus NO-Sulindac at 200 ppm. However, individual agents of Sulindac and NO-Sulindac have shown modest increases of p21^waf1/cip1^ when compared with the control diet group (Figure 4F). We also observed a significant increase in pro-apoptotic proteins expression, such as Bax (Figure 4F), and a non-significant but similar increasing trend in caspases 6/8 in rat colonic tumors administered a combination of DFMO with Sulindac or NO-Sulindac when compared to control diet (Figure 4F).

Modulation of Tumor Cell Survival Mechanism by Chemopreventive Agent Treatment. A significant decrease of β-catenin expression was observed in colonic tumors from rats fed experimental drugs, either individually or in combination, when compared to those from control group. Thus, inhibition of β-catenin expression may contribute, at least in part, to the mechanism by which DFMO and Sulindac suppress colon tumorigenesis in rats (Figure 4C,E). We observed a slight decrease in VEGF protein expression in colonic tumors from rats fed with DFMO, but a modest decrease with regard to Sulindac and NO-Sulindac. In the combination group of DFMO and NO-Sulindac, expression level of β-catenin protein was decreased when compared to control (Figure 4D,E). In addition, there was a trend toward an increase in anti-inflammatory Nrf2 expression in the drug-treated rat colonic tumors compared to control (Figure 4F). Collectively, data suggested that combinations of agents led to the modulation of various tumor driving molecular mechanisms, resulting in the prevention of colonic tumors in sporadic rat CRC model.

## 4. Discussion

We evaluated the chemopreventive efficacy of DFMO, Sulindac, and NO-Sulindac administered alone or in combination on AOM-induced colon carcinogenesis in F344 rats, which is a well-established and widely used model to develop chemopreventive agents for clinical trials [13,28,29]. Previous studies have suggested the potential usefulness of NO-NSAIDs in colon cancer prevention, as well as the possibility that they may be devoid of significant side effects [8,9,30,31,32]. Therefore, the major objective of the present study was to assess whether the derived molecule NO-Sulindac, administered alone or in combination with low-dose DFMO, would be more potent and have better efficacy than the parent compound Sulindac or a combination of Sulindac with DFMO. Our results documented, for the first time, that the low dose combination of Sulindac and DFMO significantly suppressed colon adenocarcinoma formation in AOM-treated F344 rats. The outcome of this study is of great interest, because it shows that dose de-escalation combination studies of DFMO plus Sulindac significantly suppress colonic tumorigenesis in terms of tumor incidence and multiplicity. It is also noteworthy that the degree of inhibition of colon adenocarcinoma and efficacy increased in rats administered Sulindac compared to the rats administered with NO-Sulindac.

Polyamine levels are tightly regulated by enzyme ODC and the catabolic enzyme spermidine/spermine N^1^-acetyltransferase (SSAT) in cells. ODC is highly expressed in colorectal cancer compared to adjacent normal mucosal tissue [19], and contributes to the high polyamines levels in neoplastic cells when compared to normal cells and tissues [33]. High levels of polyamines lead to rapid proliferation, while low levels lead to apoptosis and cell growth arrest [34,35,36]. As expected, there was a significant decrease in ODC activity in the rats fed DFMO, which was in line with our previous studies at higher doses (inhibition of ODC activity) [24,36], which lead to decreases in tumor volume and growth. ODC activity is not directly affected by Sulindac or NO-Sulindac, and is not statistically significant, which is in correlation with earlier studies [24]. Although there is a significant reduction in tumor volume in the combination groups, one possible reason might be that Sulindac and NO-Sulindac act on polyamine metabolism in a different manner other than the mechanism of DFMO inhibition of ODC activity, in that previous studies have shown that Sulindac induces SSAT, a gene-encoding enzyme involved in polyamine catabolism and export in colon cancer cells [37]. This is associated with a decrease in intracellular polyamine contents, which, in turn, leads to increased apoptosis [37]. Thus, Sulindac may activate polyamine catabolism and exportation [37], thereby reducing the intracellular polyamine levels and possibly acting to complement the effects of DFMO in the combination groups and significantly reducing tumor cell proliferation and increasing apoptosis. In the present study, we observed a decrease in ODC activity of the DFMO group as well as in the combination groups DFMO plus Sulindac and DFMO plus NO-Sulindac, leading to tumor growth arrest and increased apoptosis in colonic tumors.

The combined chemopreventive efficacy of Sulindac plus DFMO is higher than that of either of the compounds alone or NO-Sulindac plus DFMO. This enhanced efficacy is consistent with the notion that these compounds modulate polyamine biosynthesis synergistically. In addition to modulation of polyamine synthesis, Sulindac was associated with a significant decrease in the expression of inflammatory molecules and increased apoptosis. The role of COX-2 and iNOS in colon carcinogenesis is well established [38,39,40,41]. Importantly, iNOS has been shown to be involved in the regulation of COX-2 activity, which plays a pivotal role in colon tumorigenesis [42]. From our results, we documented that Sulindac inhibits COX-2 activity, which is consistent with our previous findings where COX-2 activity decreases with Sulindac and other NSAIDs [3]. However, the NO-Sulindac modestly decreased the COX-2 and iNOS activities, and this may be one of the reasons that the greater ability of Sulindac in combination with DFMO was found to significantly inhibit AOM-induced adenocarcinoma incidence and multiplicity in male F344 rats. It could explain the greater efficacy in preventing AOM-induced colon cancer, where the level of COX-2 in the colon has been associated with the development of tumors. A further possibility for the reduced tumor incidence and multiplicity in DFMO plus Sulindac is the additive features of both agents. The results of the present study demonstrate that inducible forms of iNOS and COX-2 are selectively inhibited in colonic tumors by DFMO plus Sulindac, contrary to the reports of others which include no change in PGE_2_ levels in the colonic mucosa of patients treated with Sulindac/DFMO combination [43]. Earlier in vitro reports show that depletion of polyamines by DFMO treatment leads to induction and posttranscriptional stabilization of COX-2 mRNA levels [44]. In contrast, we found no significant change in COX-2 activity levels in colonic tumors from rats fed DFMO. One plausible explanation for this may be that DFMO helps to stabilize mRNA but does not affect the translational process.

In addition to COX-2 and iNOS, several markers of anti-proliferation and apoptosis have been evaluated in colonic tumors that affect the pathways which play a role in the anti-tumor effects of these drugs. The role of β-catenin in colon carcinogenesis is well established, as hyperactivation of β-catenin signaling by mutations in either APC or β-catenin, leads to excessive colonocyte proliferation that leads to colon cancer development [45,46,47] and sporadic colorectal adenomas and carcinomas, which showed translocation of β-catenin from the cell membrane to the cytoplasm/nucleus [47]. Earlier reports have demonstrated that Sulindac inhibits β-catenin in human colon cancer cells [48] and in FAP patients [49]. From our observations, we found that rats fed the Sulindac diet decreased expression of β-catenin, which strongly suggests that NSAIDs exert strong tumor suppressive effects by interfering with TCF-mediated transcription. This decrease in β-catenin expression levels suggests a direct link between the tumor suppressive effects of Sulindac, which is a key defect of colorectal cancer, that deregulates *Wnt* signaling. There is not much change in β-catenin expression levels in the case of NO-Sulindac, which may be a reason for not showing much tumor inhibitory effect. Although we have no explanation for this discrepancy, the validity of our finding is strongly supported by the observation that the decrease in β-catenin expression levels may contribute, at least in part, to the mechanism by which Sulindac alone and in combination with DFMO suppresses colon tumorigenesis in rats. The chemopreventive efficacy of DFMO/Sulindac was stronger than that of either individual compound, and tumor inhibition was associated with significantly decreased cell proliferation and increased apoptosis.

We evaluated p21 and caspase markers in rat colonic tumors from different experimental groups. We observed a modest induction of p21 in colonic tumors of rats fed Sulindac and NO-Sulindac, but there was a significant induction of p21 in colonic tumors of rats fed DFMO alone or in combination with Sulindac and NO-Sulindac. It is known that ODC gene overexpression increases cellular polyamines, induces c-Myc expression, and inhibits p21^cip1/wif1^ transcription, resulting in decreased p21^cip1/wif1^ protein [50]. Results from the present study show an induction of p21^cip1/wif1^ in the DFMO treated group individually and in combination with Sulindac and NO-Sulindac. This explains the ODC activity inhibition due to the depletion of polyamine levels in the tumors, thereby decreasing c-myc expression and increasing p21^cip1/wif1^ expression, leading to tumor growth arrest. Effects of the combination of Sulindac and DFMO on tumor inhibition are mediated by stimulating apoptotic pathways by inhibiting cellular proliferation. From our study, Sulindac or NO-Sulindac alone and their combination with DFMO were able to increase the caspases 6/8, but only the combination of DFMO plus Sulindac or DFMO plus NO-Sulindac was able to increase the pro-apoptotic protein Bax in colonic tumors. However, the enhancement of apoptosis, rather than the reduction of cell proliferation, has been more consistently associated with the ability of Sulindac and NO-Sulindac to inhibit tumor growth in AOM-induced colonic tumors. The above anti-apoptotic effect coupled with downregulation of β-catenin, induction of SSAT gene, and anti-inflammatory effects of Sulindac are proven to be more potent and more effective when combined with DFMO.

## 5. Conclusions

In conclusion, our results suggest that, at the adenoma stage, administration of DFMO plus Sulindac bestowed significant adenocarcinoma inhibition with anti-inflammatory, proapoptotic, and anti-proliferative actions more efficiently than a combination of NO-Sulindac plus DFMO. Thus, the combination regimen applied in this study supports our previous low-dose combination approaches to suppress colon cancers in a synergistic and/or additive manner.

## 6. Limitations of the Study

The guidelines used here to classify the tumors are based on the WHO Classification of Tumors of the Digestive System 2010 edition (https://seer.cancer.gov/ (accessed on 1 August 2023)) (https://seer.cancer.gov/tools/solidtumor/Colon_STM.pdf (accessed on 1 August 2023)) by a clinical pathologist, rather than by a rodent histopathologist. ALT levels for all rats, including the untreated, are above the normal range, whichmay be related to the age of the rats or due to AOM treatment.

## Figures and Tables

**Table 1:** Chemopreventive effect of DFMO, Sulindac, and NO-Sulindac administered individually and in combinations on azoxymethane-induced colon adenocarcinoma formation in male F344 rats.

	Experimental Group	Colon Tumor Incidence ^#^ (Rats with Tumors/Total Rats) (% Rats with Colonic Tumors)			Colon Tumor Multiplicity ^$^ (Mean ± SEM, *N* = 30) (Mean Colonic Tumors/rat)		
		Adenoma	Adenocarcinoma	Total	Adenoma	Adenocarcinoma	Total Tumors
1	AOM/Control diet	14/30 (46.7%)	28/30 (93.3%)	29/30 (96.7%)	0.70 ± 0.18	2.80 ± 0.34	3.50 ± 0.52
2	500 ppm DFMO	12/30 (40%)	16/30 (53.3%) (*p* < 0.0009)	24/30 (80%)	0.70 ± 0.14	1.03 ± 0.22 (*p* < 0.0001)	1.73 ± 0.31 (*p* < 0.0026)
3	150 ppm Sulindac	18/30 (60%)	18/30 (60%) (*p* < 0.0048)	27/30 (90%)	0.90 ± 0.21	1.37 ± 0.29 (*p* < 0.0011)	2.27 ± 0.40 (*p* < 0.033)
4	200 ppm NO-Sulindac	19/30 (63.3%)	24/30 (80%) (*p* = 0.25)	27/30 (90%)	1.00 ± 0.22	2.16 ± 0.35 (*p* = 0.097)	3.16 ± 0.57 (*p* = 0.3)
5	500 ppm DFMO + 150 ppm Sulindac	20/30 (66.6%)	12/30 (40%) (*p* < 0.0001)	17/30 (56.6%) (*p* < 0.0004)	0.97 ± 0.22	0.53 ± 0.14 (*p* < 0.0001)	1.50 ± 0.24 (*p* < 0.0006)
6	500 ppm DFMO + 200 ppm NO-Sulindac	12/30 (40%)	15/30 (50%) (*p* < 0.0004)	21/30 (70%) (*p <* 0.001)	0.60 ± 0.16	1.06 ± 0.23 (*p* < 0.0001)	1.66 ± 0.30 (*p* < 0.0018)

^#^ Tumor Incidence—significance between the control group and treatment group was evaluated by Fisher’s Exact Test using two-tail analysis. Values are considered significant at *p* < 0.05. ^$^ Tumor Multiplicity—significance between the control group and treatment group was evaluated by unpaired *t*-test with Welch’s correction using one-tail analysis. Values are considered significant at *p* < 0.05.

**Table 2:** Effect of DFMO, Sulindac, NO-Sulindac, or combinations on AOM-induced rat colon adenocarcinoma iNOS, COX-2 and ODC Activities; and polyamine levels.

	iNOS-Activity ^a^	COX-2 Activity ^b^	ODC Activity ^c^	Polyamines Levels ^d^ (nmol/g Wet Tumor Tissue)		
				Putrescine	Spermidine	Spermine
1. Control diet	88.9 ± 7.4 ^e^	271 ± 15.3	155 ± 12.3	66.9 ± 5.8	183 ± 14	123 ± 11
2. 500 ppm DFMO	56.3 ± 5.8 ^f^ *p* < 0.005	196 ± 13.7 *p* = 0.051	82 ± 7.9 *p* < 0.001	36.3 ± 3.3 *p* < 0.001	96.3 ± 10 *p* < 0.001	67.3 ± 8.3 *p* < 0.005
3. 150 ppm Sulindac	50.6 ± 4.7 *p* < 0.001	148 ± 9.9 *p* < 0.002	128 ± 8.9 *p* = 0.2	53.7 ± 4.1 *p* = 0.09	148 ± 8.7 *p* = 0.06	128 ± 14 *p* = 0.9
4. 200 ppm NO-Sulindac	73.7 ± 5.3 *p* = 0.2	205 ± 15.5 *p* = 0.07	145 ± 13.2 *p* = 0.5	ND	ND	ND
5. 500 ppm DFMO + 150 ppm Sulindac	42.8 ± 3.5 *p* < 0.0001	122 ± 8.8 *p* < 0.0001	68 ± 4.9 *p* < 0.0001	28.9 ± 3.1 *p* < 0.0001	63.5 ± 7.2 *p* < 0.0001	49.3 ± 5.1 *p* < 0.0001
6. 500 ppm DFMO + 200 ppm NO-Sulindac	53.5 ± 4.8 *p* < 0.001	189 ± 13.1 *p* < 0.05	78 ± 9.2 *p* < 0.001	34.5 ± 3.8 *p* < 0.0005	88.5 ± 8.8 *p* < 0.0008	64.4 ± 6.8 *p* < 0.004

^a^ iNOS activity—pmol of [^3^H]-citrulline formed/mg Protein/min; ^b^ COX-2 activity—pmol of [^14^C]-15-R-HETE formed/mg Protein/min; ^c^ ODC Activity—pmol of [^14^C]-CO_2_ released from ^14^C-Ornthine/mg Protein/min; ^d^ Polyamine analysis by fluorescent HPLC system HPLC; ^e^ Mean ± SEM (*N* = 6–8); ^f^ Significantly different from control diet group, by unpaired *t*-test, with Welch’s correction. ND—not determined.

## Data Availability

The data can be shared up on request.

## Supplementary Materials

The following supporting information can be downloaded at: https://www.mdpi.com/article/10.3390/cancers15154001/s1. Figure S1. Azoxymethane-induced rat colonic tumor histologies. (a) Normal Colon (10×); (b) Adenoma (4×); (c) Adenoma (10×); (d) Adenocarcinoma (ADAC, 10×); (e,f) ADCA 40×. Figure S2: Chemoprevention of Colon Cancer by DFMO, Sulindac, and NO-Sulindac administered individually or in combinations in F344 rats Western blot replicates.

## Abbreviations

DFMO—Difluoromethylornithine; ODC—ornithine decarboxylase; AOM—azoxymethane; NSAIDS—non-steroidal anti-inflammatory drugs; AIN—American Institute of Nutrition; SSAT—spermidine/spermine N^1^-acetyltransferase; HED—Human Equivalent Dose.
