# Supplementary material for: Chemoprevention of Colon Cancer by DFMO, Sulindac, and NO-Sulindac Administered Individually or in Combinations in F344 Rats
Source: Cancers (Basel). 2023 Aug 7;15(15):4001. doi: 10.3390/cancers15154001 (PMC10417047; doi:10.3390/cancers15154001)
Supplement: Supplementary file 1 [file cancers-15-04001-s001.zip › cancers-2416679-supplementary.pdf]

Supplementary Materials

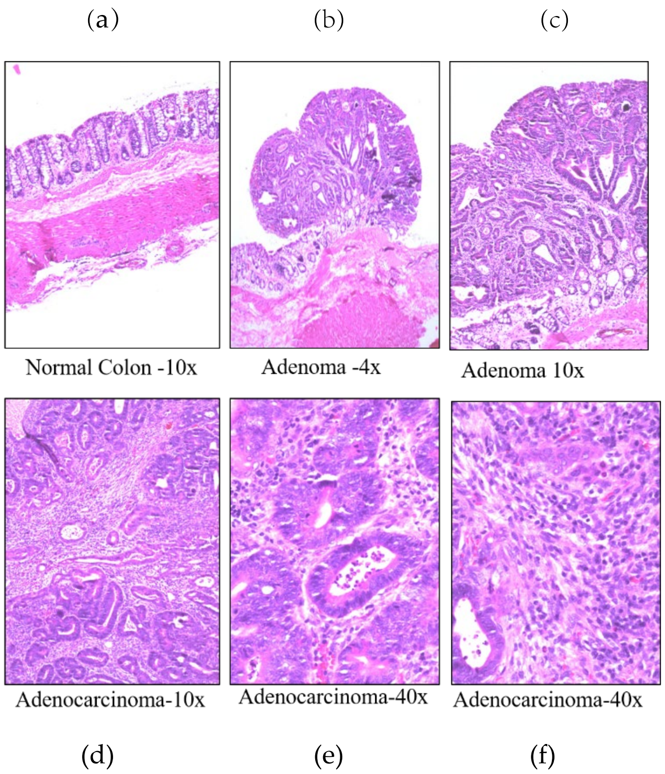

Supplementary Fgure S1. Azoxymethane-induced rat colonic tumor histologies. (a) Normal Colon (10X); (b) Adenoma (4X); (c) Adenoma (10X); (d) Adenocarcinoma (ADAC, 10X); (e,f) ADCA 40X.

Figure S2: Chemoprevention of Colon Cancer by DFMO, Sulindac, and NO-Sulindac administered individually or in combinations in F344 rats

Western blot replicates

First replicate

Caspase 6

	Tumor Control	DFMO	Sulindac	NO-sulindac	DFMO+ Sulindac	DFMO+ NO Sulindac

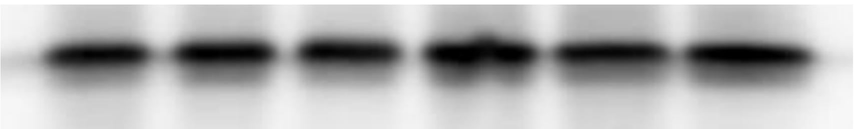

Density	11431.4	11966.89	13492.84	13868.61	11786.01	12149.23

Second replicate

Caspase 6

	Tumor Control	DFMO	Sulindac	NO-sulindac	DFMO+ Sulindac	DFMO+ NO Sulindac

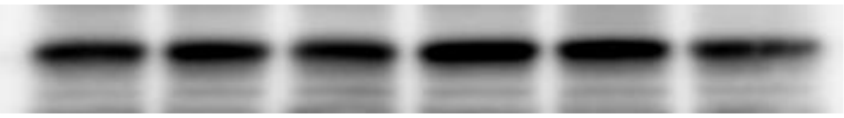

Density	15106.99	15352.04	14271.3	19095.33	16882.67	15530.35

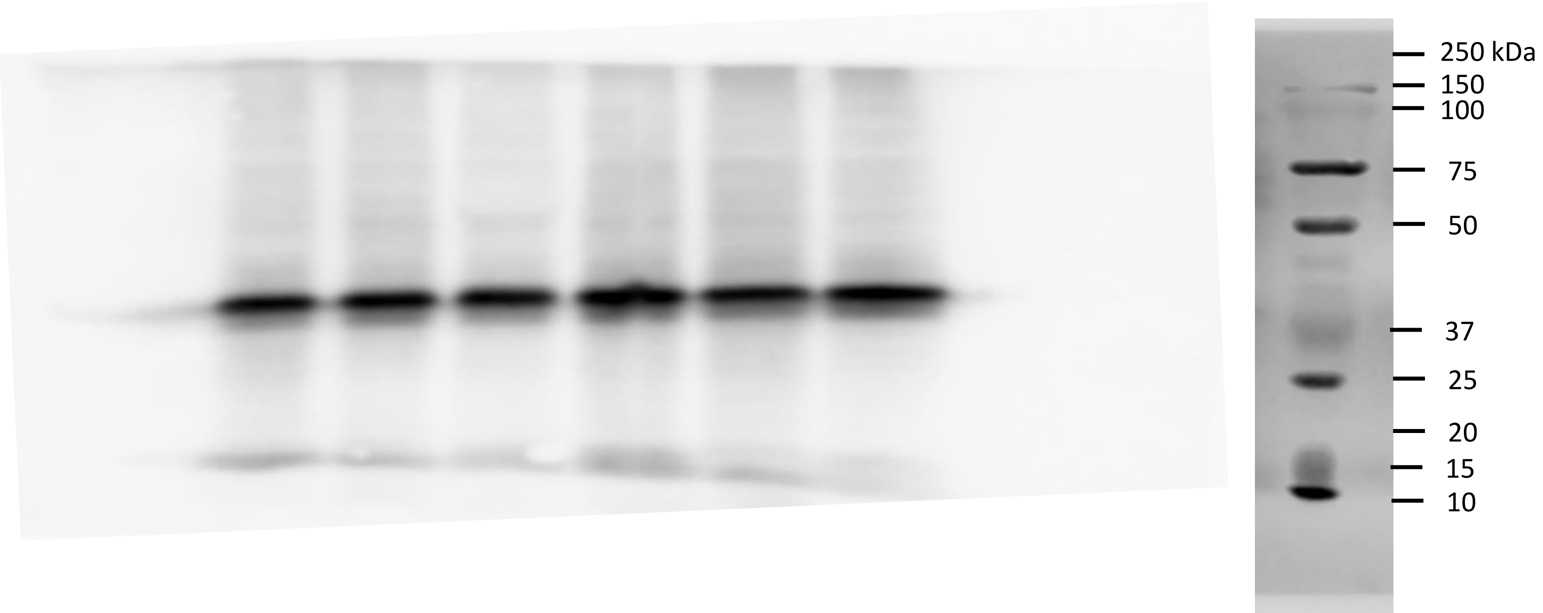

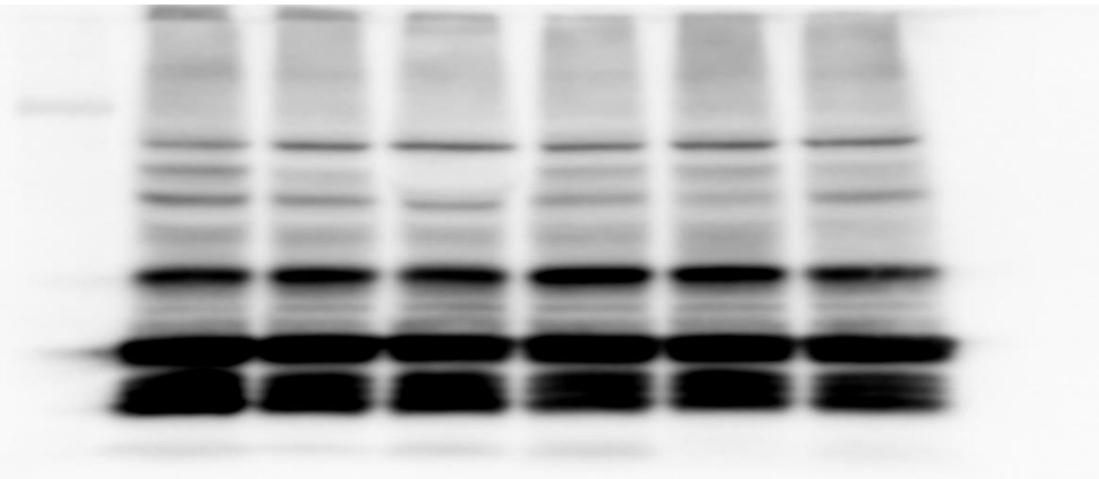

First replicate loading control

Tumor Control	DFMO	Sulindac	NO-sulindac	DFMO+ Sulindac	DFMO+ NO Sulindac

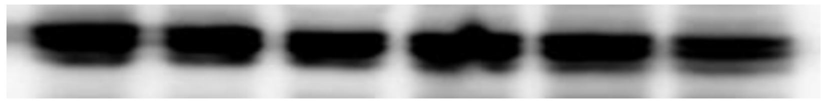

24317.16	25104.09	21126.55	18478.07	15231.51	11943.4

Second replicate loading control

Tumor Control	DFMO	Sulindac	NO-sulindac	DFMO+ Sulindac	DFMO+ NO Sulindac

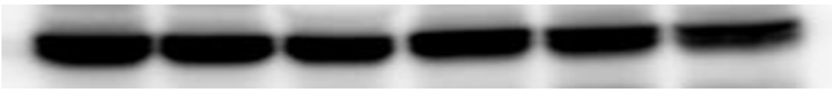

23876.92	23532.84	22540.84	24271.74	20506.92	17604.06

Loading control Normalisation	11431.4	11591.76	15530.57	18251.11	18816.4	24736.23
**Fold Change**	1	1.014028	1.358589	1.596577	1.646027	2.163884

Loading control Normalisation	15106.99	15576.5	15117.21	18784.7	19657.08	21064.28
**Fold Change**	1	1.031079	1.000677	1.243445	1.301192	1.394341

Caspase 6	Fold change					
First Replicate	1	1.014028	1.358589	1.596577	1.646027	2.163884
Seconf replicate	1	1.031079	1.000677	1.243445	1.301192	1.394341
**mean**	**1**	**1.022554**	**1.179633**	**1.420011**	**1.473609**	**1.779112**

First replicate

	Tumor Control	DFMO	Sulindac	NO-sulindac	DFMO+ Sulindac	DFMO+ NO Sulindac

Caspase 8

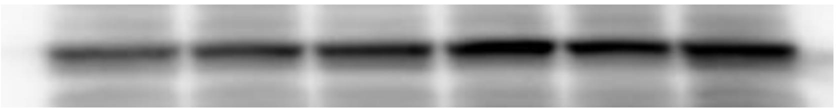

Density	9480.409	10006	12385.36	13704.7	12761.36	12750.77

Second replicate

	Tumor Control	DFMO	Sulindac	NO-sulindac	DFMO+ Sulindac	DFMO+ NO Sulindac

Caspase 8

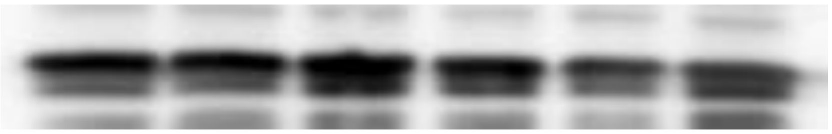

Density	22758.04	22367.77	25052.18	20789.69	16427.57	16247.16

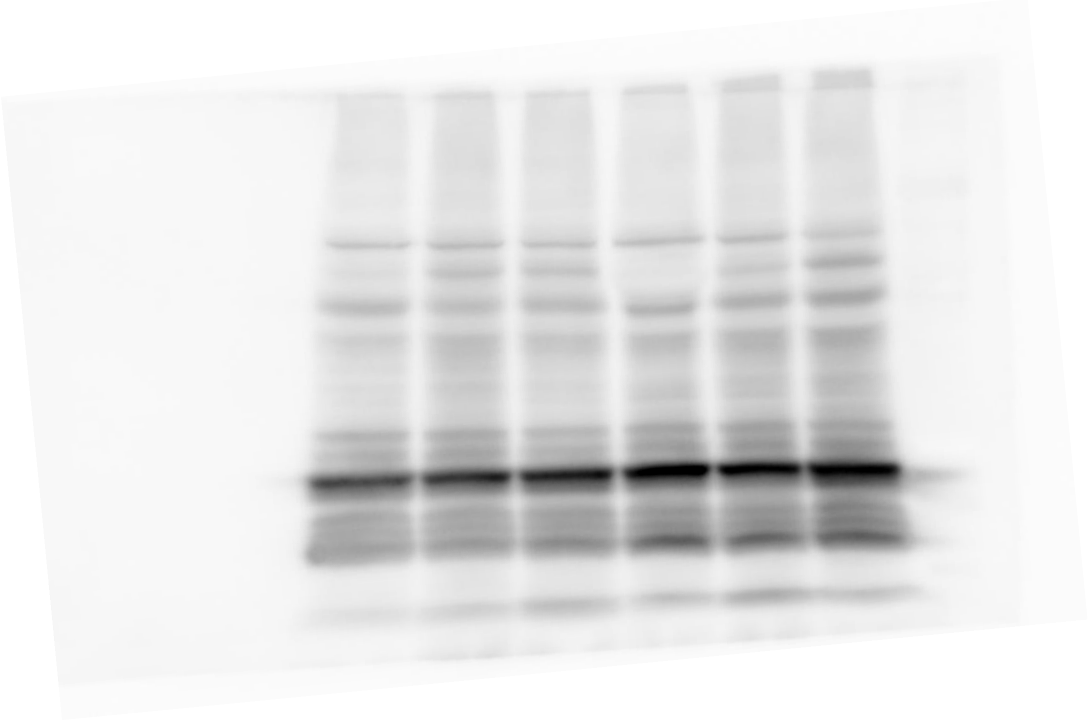

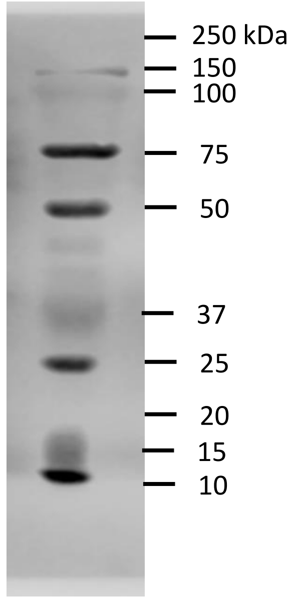

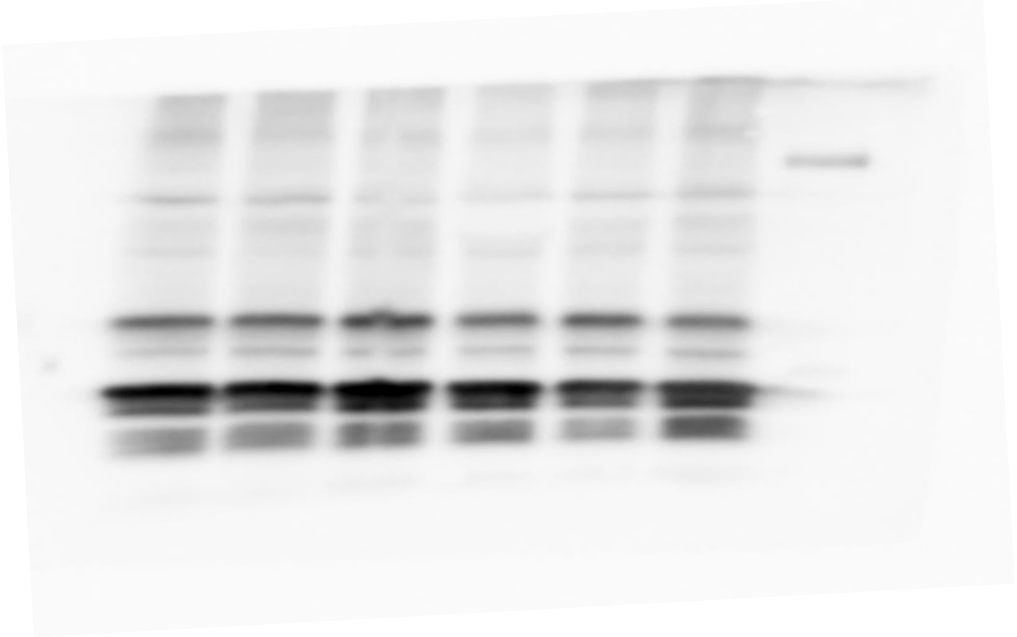

First replicate loading control

	Tumor Control	DFMO	Sulindac	NO-sulindac	DFMO+ Sulindac	DFMO+ NO Sulindac

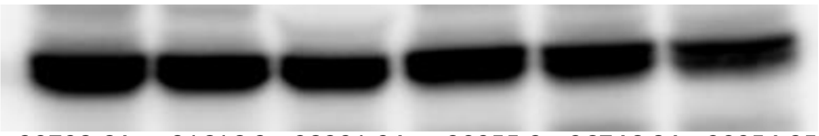

	30798.64	31616.3	28391.94	30955.3	26746.84	20954.35
Loading control Normalisation	9480.409	9747.221	13435.23	13635.35	14694.54	18741.05
**Fold Change**	1	1.028144	1.417157	1.438266	1.54999	1.976819

Second replicate loading control

	Tumor Control	DFMO	Sulindac	NO-sulindac	DFMO+ Sulindac	DFMO+ NO Sulindac

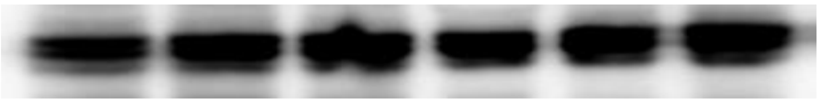

	20687.89	22269.16	25061.77	24263.28	25961.84	24013.45
Loading control Normalisation	22758.04	20779.49	20679.97	17726.16	13090.44	13997.13
**Fold Change**	1	0.913062	0.908689	0.778897	0.575201	0.615041

**Caspase 8**	Fold change					
First Replicate	1	1.028144	1.417157	1.438266	1.54999	1.976819
Seconf replicate	1	0.913062	0.908689	0.778897	0.575201	0.615041
mean	**1**	**0.970603**	**1.162923**	**1.108581**	**1.062595**	**1.29593**

First replicate

BAX

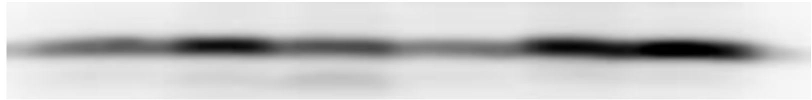

	Tumor Control	DFMO	Sulindac	NO-sulindac	DFMO+ Sulindac	DFMO+ NO Sulindac
Density	7213.489	10875.32	7970.489	6587.953	11851.39	13665.66

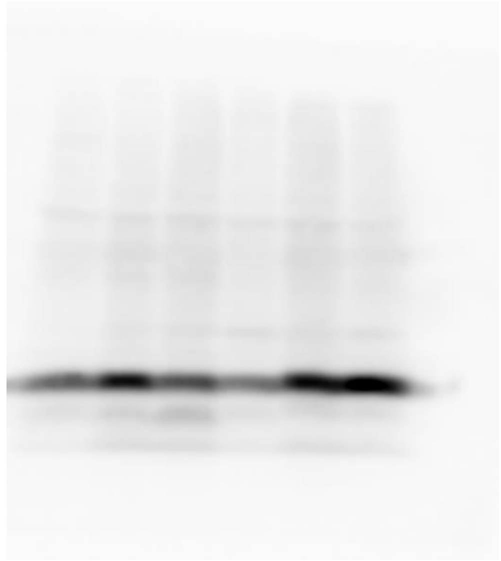

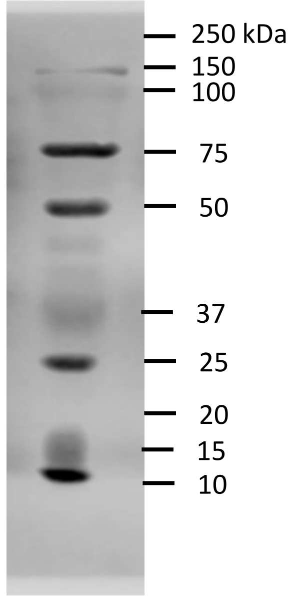

Second replicate

BAX

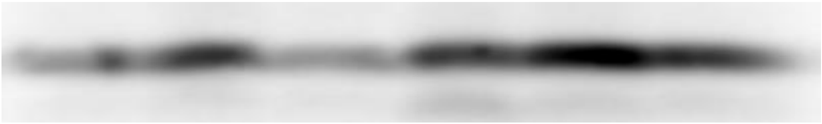

	Tumor Control	DFMO	Sulindac	NO-sulindac	DFMO+ Sulindac	DFMO+ NO Sulindac
Density	8783.288	13322.67	7292.752	13013.84	19005.84	14945.09

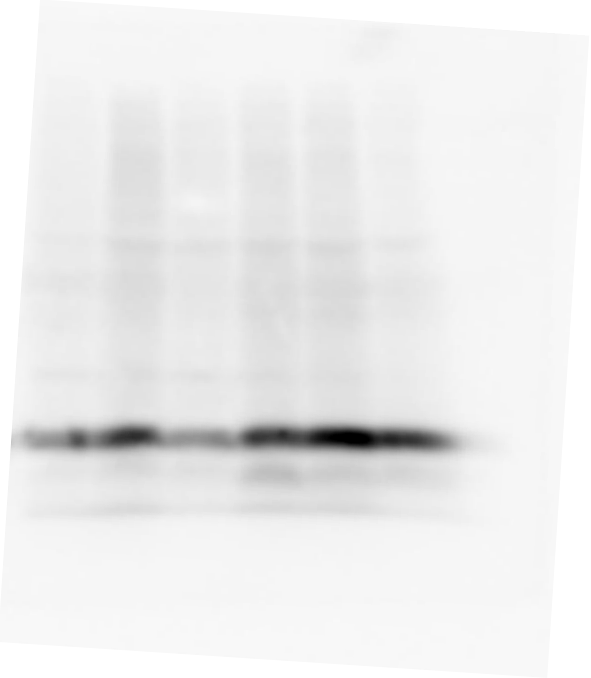

First replicate loading control

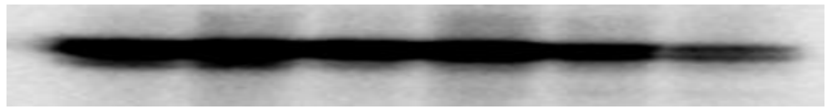

Tumor Control	DFMO	Sulindac	NO-sulindac	DFMO+ Sulindac	DFMO+ NO Sulindac
20257.17	19510.73	18518.66	16352.73	14928.73	10790.34

Loading control Normalisation	7213.489	11291.38	8718.748	8160.916	16081.44	25655.13
**Fold Change**	1	1.565315	1.208673	1.131341	2.229357	3.55655

Second replicate

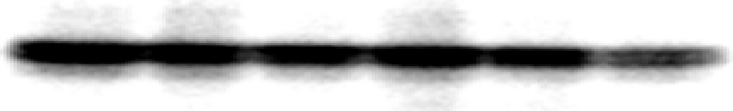

Tumor Control	DFMO	Sulindac	NO-sulindac	DFMO+ Sulindac	DFMO+ NO Sulindac
18523.58	16018.05	16105.46	14919.17	14582.56	10168.1

Loading control Normalisation	8783.288	15406.6	8387.708	16157.94	24142.28	27226
**Fold Change**	1	1.754081	0.954962	1.839623	2.748661	3.09975

BAX

	Fold change					
First Replicate	1	1.565315	1.208673	1.131341	2.229357	3.55655
Seconf replicate	1	1.754081	0.954962	1.839623	2.748661	3.09975
mean	**1**	**1.659698**	**1.081817**	**1.485482**	**2.489009**	**3.32815**

First replicate

NRF2

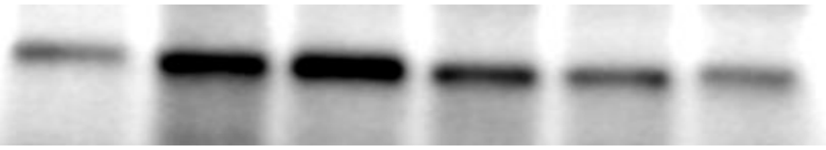

	Tumor Control	DFMO	Sulindac	NO-sulindac	DFMO+ Sulindac	DFMO+ NO Sulindac
Density	7954.187	16269.01	20975.42	11393.13	8799.915	7276.016

Second replicate

NRF2

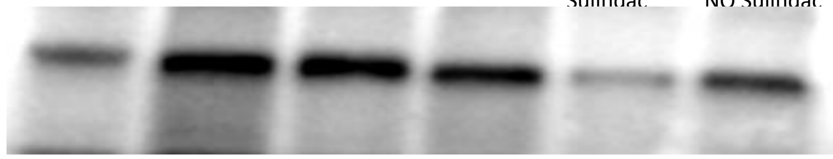

	Tumor Control	DFMO	Sulindac	NO-sulindac	DFMO+ Sulindac	DFMO+ NO Sulindac
Density	7917.53	14026.87	16575.89	13123.35	7656.032	11137.06

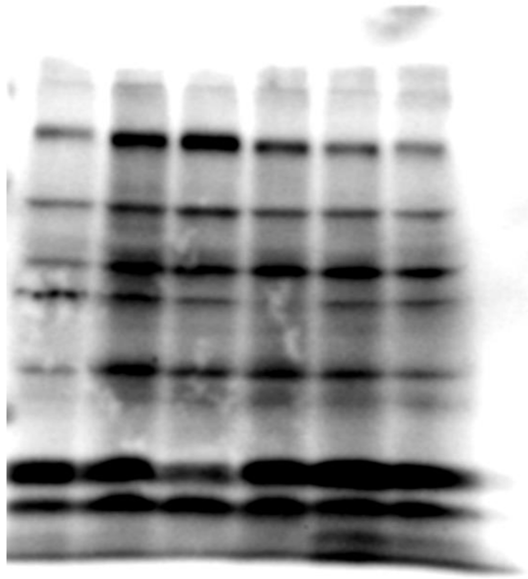

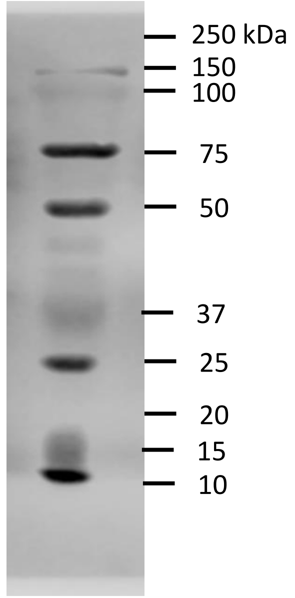

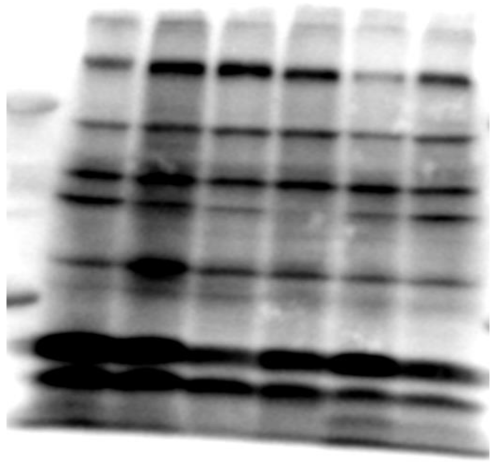

First replicate loading control

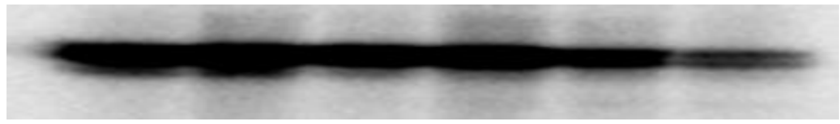

Tumor Control	DFMO	Sulindac	NO-sulindac	DFMO+ Sulindac	DFMO+ NO Sulindac
20257.17	19510.73	18518.66	16352.73	14928.73	10790.34

Second replicate loading control

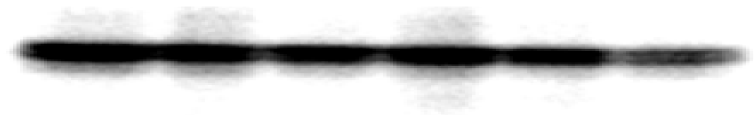

Tumor Control	DFMO	Sulindac	NO-sulindac	DFMO+ Sulindac	DFMO+ NO Sulindac
18523.58	16018.05	16105.46	14919.17	14582.56	10168.1

Loading control Normalisation	7954.187	16891.42	22944.57	14113.39	11940.82	13659.58
**Fold Change**	1	2.123589	2.88459	1.774335	1.5012	1.717282

Loading control Normalisation	7917.53	16220.94	19064.64	16293.9	9725.119	20288.77
**Fold Change**	1	2.048738	2.407902	2.057953	1.228302	2.562513

**NRF2**	Fold change					
First Replicate	1	2.123589	2.88459	1.774335	1.5012	1.717282
Seconf replicate	1	2.048738	2.407902	2.057953	1.228302	2.562513
mean	**1**	**2.086163**	**2.646246**	**1.916144**	**1.364751**	**2.139897**

p21 Revision

	Tumor Control	DFMO	Sulindac	NO-sulindac	DFMO+ Sulindac	DFMO+ NO Sulindac

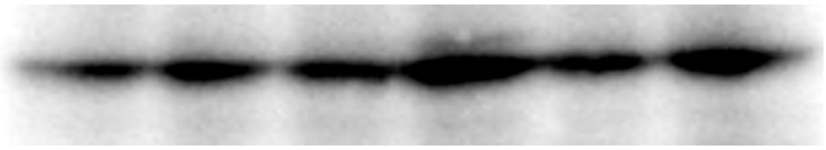

Density	15203.94	22665.15	20902.54	25827.22	22121.24	24125.09

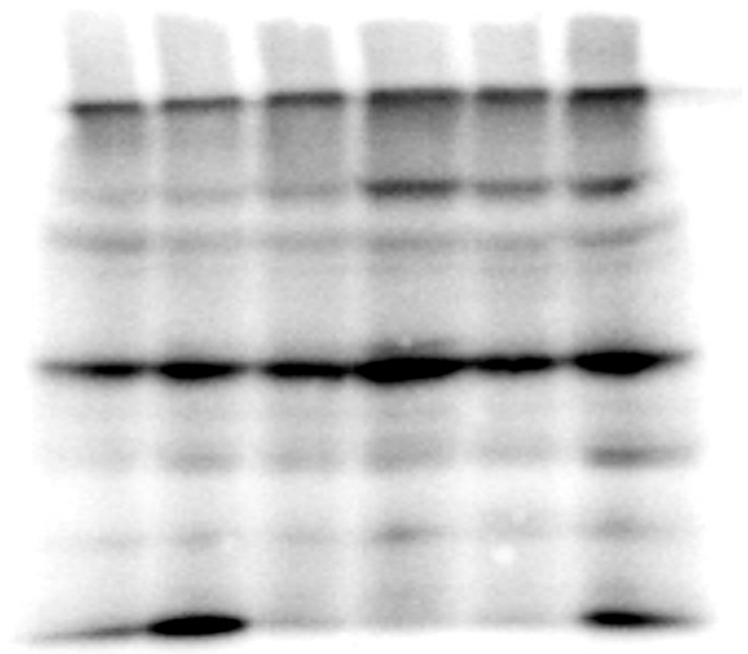

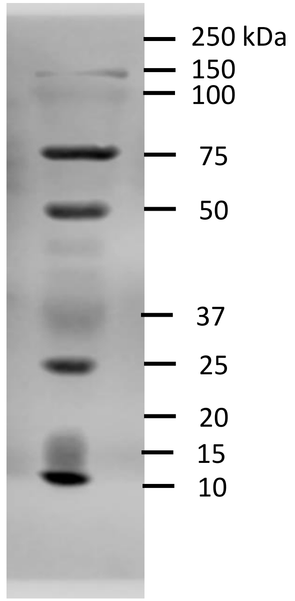

p21 Revision

	Tumor Control	DFMO	Sulindac	NO-sulindac	DFMO+ Sulindac	DFMO+ NO Sulindac

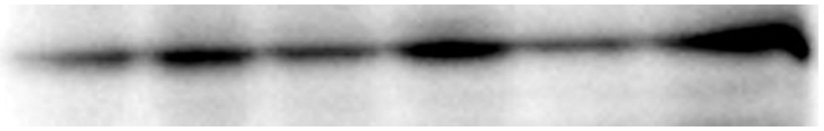

Density	11441.59	19209.46	15106.1	19477.12	11496.08	19908.85

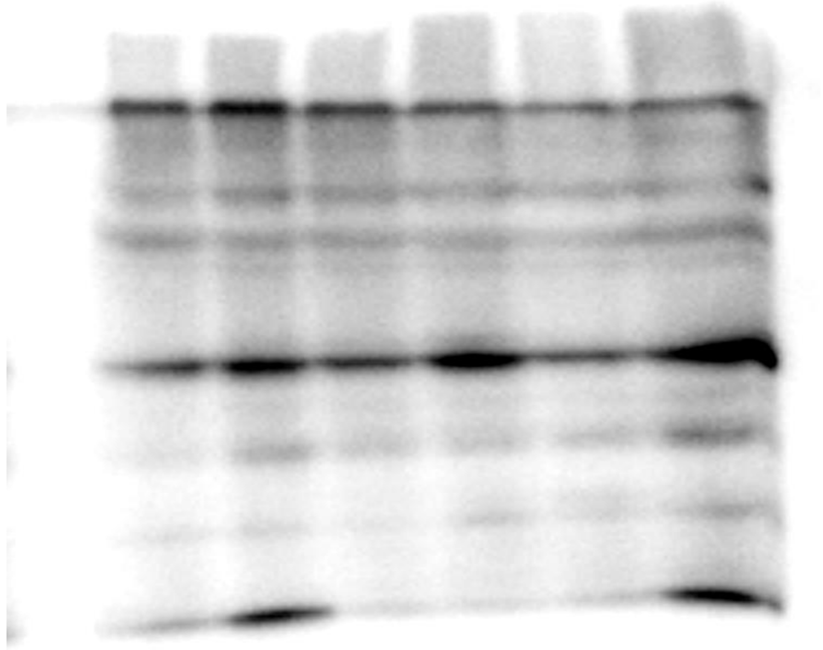

First replicate loading control

Tumor Control	DFMO	Sulindac	NO-sulindac	DFMO+ Sulindac	DFMO+ NO Sulindac

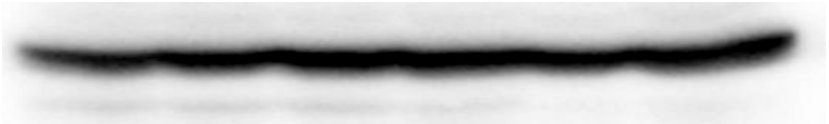

27303.16	31402.9	32559.39	34444.46	30638.64	30584.6

Second replicate loading control

Tumor Control	DFMO	Sulindac	NO-sulindac	DFMO+ Sulindac	DFMO+ NO Sulindac

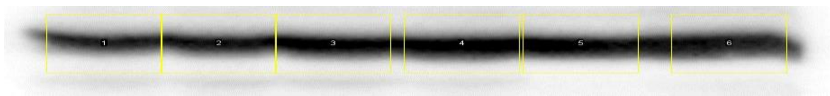

29141.04	32528.23	36246.4	37112.19	38283.51	31683.3

Loading control Normalisation	15203.94	19706.15	17528.14	20472.51	19713.01	21536.7
**Fold Change**	1	1.296122	1.152868	1.346527	1.296573	1.416521

Loading control Normalisation	11441.59	17209.17	12144.86	15293.72	8750.705	18311.36
**Fold Change**	1	1.504088	1.061466	1.336678	0.764815	1.600421

p21 Revision

	Fold change					
First Replicate	1	1.296122	1.152868	1.346527	1.296573	1.416521
Seconf replicate	1	1.504088	1.061466	1.336678	0.764815	1.600421
mean	**1**	**1.400105**	**1.107167**	**1.341602**	**1.030694**	**1.508471**
